# Effects of Engineered Nanoparticles on the Assembly of Exopolymeric Substances from Phytoplankton

**DOI:** 10.1371/journal.pone.0021865

**Published:** 2011-07-21

**Authors:** Chi-Shuo Chen, Jesse M. Anaya, Saijin Zhang, Jessica Spurgin, Chia-Ying Chuang, Chen Xu, Ai-Jun Miao, Eric Y-T. Chen, Kathleen A. Schwehr, Yuelu Jiang, Antonietta Quigg, Peter H. Santschi, Wei-Chun Chin

**Affiliations:** 1 Bioengineering, University of California, Merced, Merced, California, United States of America; 2 Department of Marine Science, Texas A & M University at Galveston, Galveston, Texas, United States of America; 3 Department of Marine Biology, Texas A & M University at Galveston, Galveston, Texas, United States of America; 4 Department of Oceanography, Texas A & M University, College Station, Texas, United States of America; 5 State Key Laboratory of Pollution Control and Resource Reuse, School of Environment, Nanjing University, Nanjing, Jiangsu Province, People's Republic of China; University of Kansas, United States of America

## Abstract

The unique properties of engineered nanoparticles (ENs) that make their industrial applications so attractive simultaneously raise questions regarding their environmental safety. ENs exhibit behaviors different from bulk materials with identical chemical compositions. Though the nanotoxicity of ENs has been studied intensively, their unintended environmental impacts remain largely unknown. Herein we report experimental results of EN interactions with exopolymeric substances (EPS) from three marine phytoplankton species: *Amphora sp.*, *Ankistrodesmus angustus* and *Phaeodactylum tricornutum*. EPS are polysaccharide-rich anionic colloid polymers released by various microorganisms that can assemble into microgels, possibly by means of hydrophobic and ionic mechanisms. Polystyrene nanoparticles (23 nm) were used in our study as model ENs. The effects of ENs on EPS assembly were monitored with dynamic laser scattering (DLS). We found that ENs can induce significant acceleration in *Amphora sp.* EPS assembly; after 72 hours EN-EPS aggregation reached equilibrium, forming microscopic gels of ∼4–6 µm in size. In contrast, ENs only cause moderate assembly kinetic acceleration for *A. angustus* and *P. tricornutum* EPS samples. Our results indicate that the effects of ENs on EPS assembly kinetics mainly depend on the hydrophobic interactions of ENs with EPS polymers. The cycling mechanism of EPS is complex. Nonetheless, the change of EPS assembly kinetics induced by ENs can be considered as one potential disturbance to the marine carbon cycle.

## Introduction

Engineered nanoparticles (ENs) are increasingly being developed to improve and innovate industrial and consumer products; for example, they are used to improve semiconductors, sunscreens and cosmetics and in the medicinal industry for imagery and drug delivery [1]. As a large fraction of atoms are located at or near their surface, ENs have high electron activities. Cytotoxic interactions between organisms and ENs can occur through various mechanisms such as electro-active groups, heavy-metal effects and reactive oxygen species (ROS) [1]. Previous studies have demonstrated the ability of algal and protozoan to uptake ENs [2]. The popularity of ENs in the consumer industry raises critical questions regarding their potential impacts on ecological systems [3], especially in the context of oceanic environments. Therefore, any EN-aquatic biota interaction that could alter natural oceanic processes, including the marine carbon cycle or marine food webs, should receive increased attention [4].

In recent years, accumulating plastic debris in the world's oceans has become a major public concern [5], [6], [7]. To date, several studies have elucidated the threat by microplastics to marine organisms such as fish, birds, and turtles —mostly through pathways of ingestion [8], [9]. Few have focused on marine phytoplankton [10] despite that fact that most floating plastic fragments—some with sizes close to 1 µm— accumulate at the ocean surface [7]; here they can degrade to leave free-floating polymers of appropriate sizes for transportation by ocean currents to neighboring regions [6], [11], [12]. These reports suggest that accumulated micropolymers may be interacting with marine phytoplankton. Though it is difficult to quantify the direct ecological influence of nanopolymers on aquatic ecosystems [13], studying the potential threat that nanopolymers released from plastic degradation [14] on ecological processes is greatly needed given reported threat by microplastics [8], [10].

Phytoplankton in the surface ocean account for about half of the global photosynthetic activity [15], making them a major driving force to sequester CO_2_ from the atmosphere [15], [16]. Furthermore, about ∼40–60% of the photosynthetic production by phytoplankton is released as EPS into the dissolved organic carbon (DOC) pool, contributing to the primary marine carbon reservoir [17], [18]. The recent discovery that ∼10% of the DOC pool can assemble to form porous microscopic gels that can be readily colonized and metabolized by marine bacteria opened a novel lens to view DOC and carbon cycling in the oceans [19], [20], [21], [22], [23]. Considering that EPS is a major source of both the marine DOC and particulate organic carbon (POC) pools [17], [18], [24], [25] understanding EPS assembly in the presence of nanoparticles and their specific mechanisms of microgel formation are critically important.

Recent studies have revealed that EN toxicity can impair phytoplankton function both extra- and intra-cellularly [26]. Miao et al. [27] found trace metal ions released from the oxidative dissolution of silver ENs in seawater were toxic to the marine diatom *Thalassiosira weissflogii*. These authors also reported that EPS production, particularly in nutrient limited cultures, played an important role in Ag ion detoxification. Zinc oxide ENs have been found to elicit a similar toxicity responses in the marine diatom *Thalassiosira pseudonana* [28]; ZnO-EN dissolution rates were accelerated in seawater, whereas ZnO-EN concentration itself only had a very small effect on Zn^2+^ release. Ag-ENs were also found to accumulate inside the freshwater alga *Ochromonas danica* where they exerted their toxic effects [29].

Here we used EPS released by three phytoplankton—*Amphora sp.*, *A. angustus* and . *tricornutum*—to investigate the effects of ENs on EPS assembly. *Amphora sp.* is a major genus of diatoms that has a world-wide distribution and an ability to grow under a wide range of conditions [30]. *Amphora sp.* is also a dominant fouling/biofilm diatom species that produces significant amount of EPS [30] and has been used in many diatom mobility studies [31], [32]. *Ankistrodesmus* is a major genus of green algae that has been used in many studies [33], [34]. *P. tricornutum* is a model diatom for genomics [35], [36] and fatty acid metabolism studies [37]. To investigate the environmental impacts of nanoplastics released during debris degradation, polystyrene ENs (diameter 23 nm) were used as model ENs. Their high surface ratio and nano-scale particle size provided a suitable model to study the interactions of engineered nanomaterials and natural polymers. In this study, particle sizing by dynamic laser scattering (DLS) was used to monitor the assembly process of EPS and their interactions with ENs. Hydrophobic dye (Nile Red) and protein∶carbohydrate ratios were applied to quantify the existence of hydrophobic domains on EPS polymers and to investigate the role hydrophobic interactions in EN-induced EPS assembly [38].

## Materials and Methods

### Chemicals and solution preparation

HPLC grade reagents and salts including sodium chloride, potassium chloride, calcium chloride, magnesium chloride, magnesium sulfate, sodium bicarbonate, and dimethyl sulfoxide were purchased from Sigma-Aldrich (St. Lious, MO, USA). Polystyrene nanoparticles (Bangs Laboratories, IN, USA) were used in our study as model ENs. The primary size and surface area of these non-fluorescence nanoparticles were 23 nm and 2.48×10^14^ µm^2^/g (certificate provided by vendor). Fluorescence polystyrene ENs (23 nm, Bangs Laboratories, IN, USA) were used only for fluorescence microscopy. The size of these ENs was independently confirmed by DLS (dynamic laser scattering) in lab. Thorough sonication was applied to nanoparticle stock solutions before experiments, as described in our previous study [39].

Artificial Seawater (ASW, 423 mM NaCl, 9 mM KCl, 9.27 mM CaCl_2_, 22.94 mM MgCl_2_, 25.5 mM MgSO_4_, 2.14 mM NaHCO_3_) was prepared using deionized water from a Milli-Q system (Millipore, Billerica, MA, USA) following established protocols from the Marine Biological Laboratory, Woods Hole, MA. The composition of Ca^2+^- free ASW included: 436.7 mM NaCl, 9 mM KCl, 22.9 mM MgCl_2_, 25.5 mM MgSO_4_, 2.1 mM NaHCO_3_, and 1 mM EGTA. Two different concentrations of ENs were added into the EPS solution (final EN concentration: 10 ppb, 100 ppb). A Nile Red stock solution (1.6 mM) was prepared in DMSO. A Chlortetracycline hydrochloride (CTC) (Sigma-Aldrich, USA) stock solution (10 mM) was prepared in Milli-Q water.

### Extraction of exopolymeric substances (EPS) from phytoplankton culture

*A. angustus* and *P. tricornutum* used for EPS extraction were purchased from the CCMP (The Provasoli-Guillard National Center for Culture of Marine Phytoplankton). Both species were grown under continuous light (35 µmol. photons m^−2^ s^−1^) at 20°C. The culture volume was 20 L for each and cultures were aerated. Growth was monitored by measuring the change in optical density at 750 nm with a UV/VIS spectrophotometer. Cultures were harvested during the stationary phase and then EPS was extracted. The phytoplankton culture was centrifuged at 3200 rpm for 30 minutes, after which it was separated into pellet (cells) and supernatant fractions. The supernatant fraction was used to collect free dissolved EPS according to the previous study [40]. In brief, the procedure consisted of a) filtration, b) cross-flow ultrafiltration, c) stirred-cell diafiltration. 20 L of the pre-filtered supernatant fraction (<0.45 µm) was ultrafiltered until 200–300 mL of retentate was left. The cartridge was rinsed with 200 mL of pure water and soaked for 6 hours. After that, the cartridge was rinsed with another 200 mL of water, and this whole process was repeated twice. Subsequently, the retentate solution and four rinse solutions were combined. The resulting 1 L of solution was then further concentrated by stirred-cell diafiltration with a 5 kDa membrane to obtain 50 mL of concentrated EPS solution. EPS of *Amphora sp.* was obtained using the same approach described by Zhang et al. [41].

### Compositional characterization of EPS

Carbohydrate concentration was measured using the anthrone method [40], with glucose as a standard. Uronic acids were determined according to Blumenkrantz and Asboe-Hansen with glucuronic acid as a standard [42]. Proteins were measured using a modified Lowry Protein Assay Kit (Pierce, 23240, USA), according to the protocol provided by the manufacture. Additionally, elemental carbon, hydrogen and nitrogen abundance was analyzed by Series II CHNS/O Analyzer 2400 (Perkin Elmer).

### Estimation of EPS molecular weight

Size Exclusion Chromatography (SEC) was used to measure the molecular weight of the EPS [41]. Briefly, 150 µL of EPS solution was injected into a Tosoh TSK G-4000PW×l (300×7.8 mm) and detected by a Refractive Index detector. The mobile phase was 0.078 M NaNO_3_ in 10 mM phosphate buffer (pH = 6.8) at a flow rate of 0.5 mL/min. Polystyrene standards with a molecular weight of 8 kDa, 35 kDa, 100 kDa, and 780 kDa were used for establishing the calibration curve, whereby the logarithm of molecular weight was plotted vs. corresponding retention time.

### Microgel Sizing

EN-induced alterations of EPS assembly were investigated with three different types of EPS (*Amphora sp.*, *A. angustus* and *P. tricornutum*) and at three EN concentrations (0, 10, 100 ppb) in ASW and Ca^2+^-free ASW. The size of assembled EPS gels (microgels) was monitored by DLS following protocols published previously [19]. The EPS solution was briefly shaken, and refiltered through a 0.22-µm Millipore membrane (pre-washed with 0.1N HCl) before use. Aliquots were then poured directly into scattering sample vials. Scattering cells were positioned in the goniometer of a Brookhaven laser spectrometer (Brookhaven Instruments, Holtsville, NY, USA). EPS assembly was monitored for two weeks, by analyzing the scattering fluctuations detected at a 45 degree scattering angle. The autocorrelation function of the scattering intensity fluctuations was averaged over a 12-min sampling time, using a Brookhaven BI 9000AT autocorrelator. CONTIN method was adapted to calculate particle size distribution [19], [43]. Calibration of the DLS method was conducted using standard suspensions of latex microspheres (Polysciences, Warrington, PA, USA). Each measurement was taken in replicate (n = 6) at room temperature.

### Fluorescence enhancement measurement

Nile Red (Invitrogen, Carlsbad, CA, USA), used as a hydrophobic indicator as in our previous studies [38], is a particularly effective solvatochromic dye containing a rigid aromatic group and an exocyclic diethylamine group. The absorbance and fluorescence emission depends on the physical properties of surrounding solvent environment: fluorescence emission is enhanced with hydrophobic environment exposure [44]. EPS from *Amphora sp.*, *A. angustus* and *P. tricornutum* were mixed with 13 µM Nile Red in triplicate. The fluorescence measurements were obtained with a Shimadzu RF-5000U spectrofluorophotometer (λ_excitation_ = 550 nm; λ_emission_ = 633 nm). Fluorescence emission of Nile Red showed a very weak signal at 633 nm in the polar ASW with excitation wavelength 550 nm.

CTC was used to reveal bound Ca^2+^ on EPS polymers [19], [45]. CTC (100 µM) was added into ASW mixed with each type of EPS. For CTC fluorescence measurements, the emission was collected at λ = 530 nm (excited at λ = 390 nm) using a Shimadzu RF-5000U spectrofluorophotometer.

### Environmental scanning electron microscopy (ESEM)

ESEM was used to investigate EPS polymer networks in their native conformations. This method provides a non-destructive tool to study materials at electron microscopy resolution while still fully hydrated. Samples of EPS were prepared in ASW with/without 23 nm polystyrene nanoparticles, as previously described [19], [20], [21], [22], [23]. After being incubated for 10 days in darkness to reach the equilibrium sizes, all EPS aggregations were filtered through a 0.22-µm Millipore Isopore membrane (Fisher Scientific, Pittsburgh, PA, USA). The assembled microgels retained on filters were investigated using FEI Quanta 200 ESEM (North America NanoPort, Portland, OR, USA).

### Fluorescence microscopy

The accumulation of ENs within EPS microgels was investigated with Fluorescence Microscopy (Nikon Instruments, Melville, NY, USA). EN-induced EPS microgels was prepared in agreement with our aforementioned protocol with 100 ppb 23 nm fluorescent ENs (Bangs Laboratories, IN, USA) and 13 µM Nile Red. The fluorescent images of microgels retained on Isopore membrane were collected by fluorescence microscopy with at λ_excitation_ = 530 nm (Nile red) and at λ_excitation_ = 488 nm (fluorescent ENs).

### Statistical analysis

Data represent means ±1 standard deviation (SD). Each experiment was performed in triplicate. A student's t-test analysis was used to determine statistical significance. p values of <0.05 were used as standard for statistical significance (GraphPad Prism 4.0, GraphPad Software, San Diego, CA).

## Results

### Chemical analysis of EPS

EPS from the three phytoplankton species contained varied protein∶carbohydrate ratios, which can be taken as an indicator of their relative hydrophobicity. *A. angustus* had a ratio of 0.72, *P. tricornutum* with a ratio of 0.31 while *Amphora sp.* had no detectable level of proteins (Table 1, Zhang et al 2008). Our chemical analysis indicates that EPS from these three phytoplankton species have similar molecular weight distributions and uronic acid ratios (Table 1). CHN analysis also indicated that *Amphora* EPS has the lowest N content of the three types of EPS (Table 1). Of the different EPS used in this study, EPS from *A. angustus* is the most hydrophobic whilst *Amphora* EPS is the most hydrophilic.

**Table 1:** Chemical analysis of EPS.

Marine Phytoplankton	Molecular weight distribution (kDa)	Protein/carbohydrateRatio	Uronic acid/carbohydrate Ratio	C%	H%	N%
*Amphora sp.* *	1000.0	∼0	0.5	37.7	6.27	1.37
*Ankistrodesmus angustus*	1026.7, 123, 13.2, 2.6	0.72	0.48	41.8	7.34	5.83
*Phaeodactylum tricornutum*	1005.9, 126.4, 36.1, 22.7, 12.8	0.31	0.5	37.6	5.71	4.5

*Zhange et al., (2008) [40].

### Assembly of EPS from phytoplankton in ASW and Ca^2+^- free ASW

**Figure 1:**
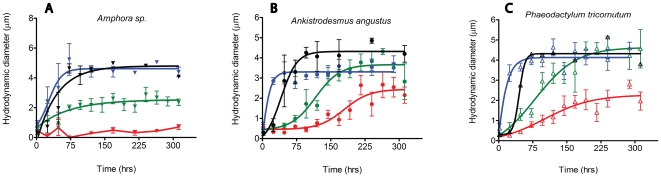
Assembly kinetics of EPS monitored with DLS. (A) Assembly kinetics of EPS of *Amphora sp.* (B) Assembly kinetics of EPS of *Ankistrodesmus angustus* (C) Assembly kinetics of EPS of *Phaeodactylum tricornutum* EPS assembly in Ca^2+^-free ASW (black) was monitored to investigate assembly kinetics with decreased divalent ion availability. Different concentrations of ENs (polystyrene nanoparticles): 0 (red), 10 (green) and 100 ppb (blue), were added to investigate the effect of ENs on EPS microgel formation.

The spontaneous assembly of 100 µg L^−1^ *Amphora sp.* EPS solutions in ASW containing 9 mM Ca^2+^ (but no ENs) was monitored by DLS for more than 10 days. As shown in Fig. 1a, EPS from *Amphora sp.* cannot assemble to form EPS microgels after 10 days. Similar measurements conducted in 100 µg L^−1^ *Amphora sp.* EPS solutions with ENs (10 or 100 ppb) demonstrated that ENs can facilitate EPS assembly following first-order kinetics, reaching steady-state assembly/dispersion equilibrium in ∼60 hrs. The equilibrium size of microgels, ∼2.5 µm formed with 10 ppb ENs, was significantly smaller than those formed from100 ppb ENs (4–5 µm) (Fig. 1a).

The same protocol was used to test the spontaneous assembly of EPS from *A. angustus* (Fig. 1b) and *P. tricornutum* (Fig. 1c). Results show that both types of EPS polymers can assemble following almost identical kinetics and reach similar microgel equilibrium sizes (∼2 µm) in the absence of ENs. With 10 ppb ENs, for both EPS types, the assembly rate was accelerated and the resulting microgel size was increased to around 4 µm (Fig. 1b, c). With higher concentrations of ENs (100 ppb), the assembly rate was further accelerated; however, the resulting microgel size remained at around 4 µm (Fig. 1b, c). We also monitored pure ENs in ASW for 10 days with DLS and found no EN self-aggregation (data not shown).

The EN-induced EPS assemblies from those three species were monitored with 100 ppb ENs in Ca^2+^-free ASW. Without divalent ions (Ca^2+^ or Mg^2+^), our results show ENs still can promote EPS assembly. EPS from *Amphora sp*, *A. angustus* and *P. tricornutum* assembled into microgels with equilibrium sizes about 4–5 µm within ∼120 hrs (Fig. 1a–c).

**Figure 2:**
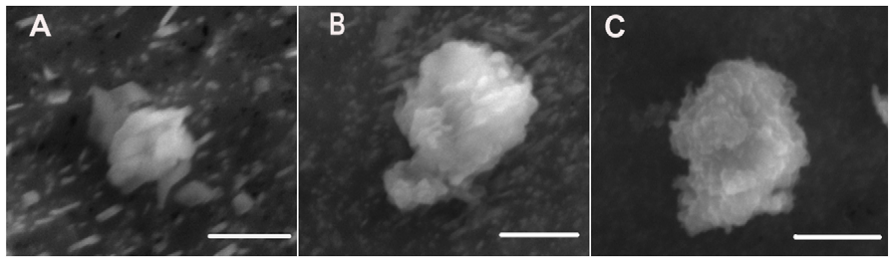
ESEM images of EPS microgel. (A) *Amphora sp.* (Scale Bar = 4 µm) (B) *Ankistrodesmus angustus* (Scale Bar = 5 µm) (C) *Phaeodactylum tricornutum* (Scale Bar = 5 µm).

**Figure 3:**
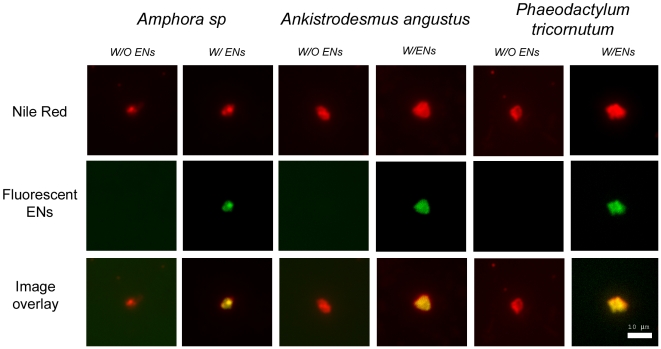
Fluorescence images of EPS and ENs-induced EPS microgels. Nile Red was used to determine the microgel morphology. Green fluorescent signals indicated the fluorescent ENs. From the overlay images, results showed that the ENs incorporated within EPS matrixes. Scale bar is 10 µm.

ESEM images (Fig. 2a–c) showed that ENs (100 ppb) may incorporate into EPS microgel matrices, as granular surface structures were found in these ESEM images (Fig. 2a–c). In addition, ESEM observations confirmed the size measurements with DLS (Fig. 1a–c). The results from fluorescence microscopy supported the hypothesis that ENs may be incorporated into EPS microgels. In our experiments, EPS microgels were stained with Nile Red to determine the gel morphology (Fig. 3). Noting EPS assembly in ASW without ENs, green fluorescent ENs were found in EPS matrices of all three species (Fig. 3). The data indicate that ENs can accumulate within EPS polymer matrices and may reach a higher concentration than the bulk EN concentration in the water column.

### Fluorescence of Nile Red and CTC

We used Nile Red, a widely used hydrophobic fluorescent probe, to detect the presence of hydrophobic regions in EPS from *Amphora sp.*, *A. angustus* and *P. tricornutum* (Fig. 3). The higher Nile Red fluorescence intensity observed in EPS from *A. angustus* and *P. tricornutum* indicated these EPS are much more hydrophobic than EPS from *Amphora sp*. The Nile Red fluorescence results here are consistent with chemical analysis results (Table 1) demonstrating that EPS from *A. angustus* is the most hydrophobic and *Amphora sp.* EPS is the least hydrophobic.

**Figure 4:**
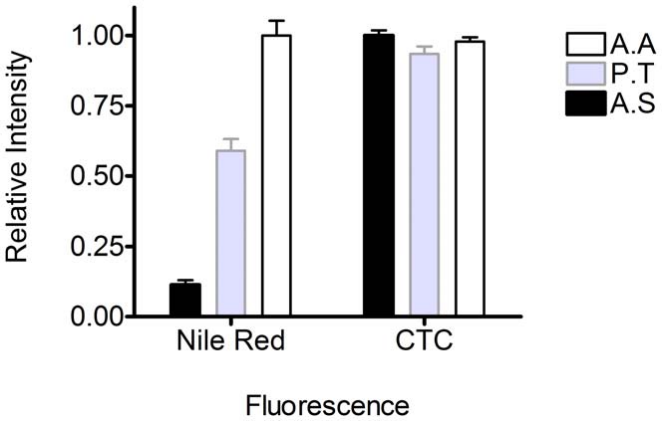
Fluorescence measurements of Nile red and CTC for EPS. EPS extracted from various phytoplankton, *Amphora sp.* (black), *Phaeodactylum tricornutum* (grey) , and *Ankistrodesmus angustus* (white).

CTC has been used to monitor bound Ca^2+^ in marine microgels [19], [45]. CTC fluorescence indicates that all three types of phytoplankton EPS have similar Ca^2+^ binding capacity (Fig. 4).

## Discussion

Both hydrophobic and electrostatic interactions have been demonstrated to play important roles in the assembly of EPS microgels [23], [38]. Our results indicate that only 10 to 100 ppb of ENs released to the aquatic environment can cause significant EPS-assembly changes (Fig. 1a–c). The results from *A. angustus* and *P. tricornutum* show that ENs can accelerate the assembly kinetics and increase the equilibrium microgel sizes of EPS. For EPS from *Amphora*, ENs can effectively induce their assembly, resulting in microgels with an equilibrium size of 4–6 µm, similar to *A. angustus* and *P. tricornutum* EPS.

EPS polymers from marine organisms are polysaccharide-rich, containing uronic acids and various proteins. Slight changes in their compositions would therefore affect their physic-chemical, e.g., biosurfactant and emulsifying, properties. Their role and fate in biogeochemical cycles is largely unexplored [46], [47]. Generally, the acidic groups in EPS are carboxylate, sulphate, and phosphate. Protein constituents have been proposed to be the major contributor for the hydrophobic domains of EPS [38], [48].

Different protein∶carbohydrate ratios were found in our chemical analysis of EPS (Table 1). Nile Red and CTC were used to detect the presence of hydrophobic domains and Ca^2+^ binding on different EPS polymers [19], [23], [38], [45]. Our Nile Red fluorescence results indicate greater hydrophobic domains on EPS polymers with higher protein∶carbohydrate ratios, which are in agreement with previous reports showing protein content contributes to amphiphilic and emulsifying properties of hydrocolloids such as EPS [49], [50]. Spontaneous assembly was only observed with EPS from *A. angustus* and *P. tricornutum*, which have higher protein∶carbohydrate ratios, and not from *Amphora sp.* EPS (Fig. 1a–c), which has a low protein∶carbohydrate ratio (i.e., no detectable protein, lack of sufficient hydrophobic domains, Table 1). Our data indicate that the hydrophobic domains on EPS polymers potentially serve as the essential aggregation sites for EPS assembly (Fig. 1a–c) [51]. For EPS with a relatively low protein (hydrophobic) fraction, such as *Amphora sp.*, 100 ppb ENs is required to trigger significant EPS assembly. However, for both partially hydrophobic EPS from *A. angustus* and *P. tricornutum*, 10 ppb ENs are sufficient to significantly change assembly kinetics, resulting in larger microgels (4–5 µm) (Fig. 1b–c). Our data thus confirm the importance of hydrophobic interactions and the total amount of EPS, as well as indicate the threshold of released nanowaste that can disturb the EPS assembly, which may be related to the specific protein∶carbohydrate ratio of EPS.

The Ca^2+^ -free ASW data further confirmed that hydrophobic interactions play a critical role in phytoplankton ENs-induced EPS assembly (Fig. 1). With a lack of divalent ions serving as cross-linkers, similar EN-induced EPS assembly was observed in 100 ppb EN concentrations. Our results provide evidence that hydrophobic interactions play critical roles in the assembly of these marine EPS, consistent with the previous findings [23].

The chemical analysis in our study indicates that EPS from these three phytoplankton species have similar molecular weight distributions (*Amphora*: 1000 kD; *A. angustus*: 1028 kD; *P. tricornutum*: 1006 kD) (Table 1). These observations are consistent with the predictions from a previous polymer model that states the equilibrium size of entangled polymer matrices depends on the polymer length [52], [53]. Due to the heterogeneous chemical compositions, the mechanism of EPS assembly remains elusive. In order to quantify the accelerations effects of ENs, the assembly kinetics was fitted with a sigmoidal curve and T_1/2_ was used to represent the time needed for the microgel to reach half of the equilibrium size. *A. angustus* showed a dramatically decreased T_1/2_: from ∼178 hrs (control, without ENs) to ∼16 hrs with 100 ppb ENs. Similar positive correlations between T_1/2_ decrease and ENs dosages were found in *P. tricornutum* and *Amphora sp.* EPS (Table 2). The acceleration of assembly kinetics shown here demonstrated that ENs are able to promote EPS assembly. In addition, we used Hill coefficients to investigate the cooperative interactions between ENs and EPS polymers [51] . For *Amphora* EPS, the Hill coefficient was ∼2.28 for 100 ppb ENs concentration. Positive EN-EPS cooperative effects were also observed in EPS assembly of *A. angustus* and *P. tricornutum* (Table 2). Similar positive cooperation was also found to correlate with hydrophobic interactions between ENs and EPS.

**Table 2:** EPS Assembly analysis.

Phytoplankton	Amphora sp.*			*Ankistrodesmus angustus*			*Phaeodactylum tricornutum*		
ENs concentration (ppb)	*N.A*	10	100	*N.A*	10	100	*N.A*	10	100
T_1/2_ (hrs)	–	46	28	*178*	116	16	119	96	16
Hill coefficient	–	0.84	2.28	–	1.97	1.33	–	2.38	1.46

EPS play critical roles in aquatic ecosystems and have been shown to be key sources for marine DOC and POC. If the EN-induced changes of EPS assembly highlighted in this study are applicable to the natural environment, ENs can lead to deleterious environmental impacts. With varying assembly characteristics, EPS released from diverse phytoplankton contribute to different organic carbon pools in the ocean. Our data reported here indicate that 10 ppb ENs can unexpectedly induce assembly of EPS of *Amphora sp.*, indicating the re-direction of the organic carbon flux from the DOC to POC pool. The alterations of EPS assembly kinetics from *A. angustus* and *P. tricornutum* also indicate the change of the time scale for carbon flow between DOC and POC.

EPS assembly changes can also affect the microbial ecosystem and marine trophic cycle. Described as the dark matter of biofilms and transparent exopolymeric particles (TEP), EPS play crucial roles in the formation and maintenance of structured multicelluar microbial communities [21], [54], [55], [56], [57], [58], [59], [60]. The concentration, cohesion, charge, sorption capacity, specificity and nature of the individual components of EPS, as well as the three-dimensional architecture of the matrix (the dense areas, pores and channels), determine the mode of community life. In this study, our data showed that ENs can drastically change the assembly behavior of EPS. The change of sedimentation velocity, caused by assembly size changes, can reshape the plume leaking from microgel and influence the nutrient utilization of free-living microbes in the water column [54], [61]. Moreover, higher concentrations of ENs accumulated within EPS microgel matrices were shown with ESEM and fluorescence microscopy (Fig. 2a–c). Because microgels also serve as important nutrition source for the marine food web in the deep ocean, the possibility of ENs impacting direct up-take by higher-level organisms, such as protozoa and metazoan, needs to also be considered [62].

Our data thus show the EPS gel matrices serve as a concentrating sponge (Fig. 3). This new pathway of nanowaste accumulation facilitated by EPS suggests an urgent need to consider lower concentration limits for nanowaste in marine environments [3], [63]. In summary, our results clearly demonstrate how nanowaste (e.g. nanoparticles) can potentially disturb the marine carbon cycle and ecosystem [58]. Whereas most environmental impact studies of nanomaterials have focused on “nanotoxicity”—investigating the direct harmful effects on phytoplankton cells or various organisms—our study indicates that indirect influences from ENs can potentially pose greater environmental threats than those of direct toxicity.
